# Rolling up transition metal dichalcogenide nanoscrolls via one drop of ethanol

**DOI:** 10.1038/s41467-018-03752-5

**Published:** 2018-04-03

**Authors:** Xueping Cui, Zhizhi Kong, Enlai Gao, Dazhen Huang, Yang Hao, Hongguang Shen, Chong-an Di, Zhiping Xu, Jian Zheng, Daoben Zhu

**Affiliations:** 1 Beijing National Laboratory for Molecular Sciences, Key Laboratory of Organic Solids, Institute of Chemistry, CAS, Beijing, 100190 China; 2 University of Chinese Academy of Sciences, Beijing, 100049 China; 3 Department of Engineering Mechanics and Center for Nano and Micro Mechanics, Applied Mechanics Laboratory, Tsinghua University, Beijing, 100084 China

## Abstract

Two-dimensional transition metal dichalcogenides (TMDs) have attracted lots of interest because of their potential for electronic and optoelectronic applications. Atomically thin TMD flakes were believed capable to scroll into nanoscrolls (NSs) with distinct properties. However, limited by mechanical strength and chemical stability, production of high-quality TMD NSs remains challenging. Here, we scroll chemical vapor deposition-grown monolayer TMD flakes into high-quality NSs in situ in 5 s with a nearly 100% yield by only one droplet of ethanol solution. An obvious photoluminescence is demonstrated in NSs and the self-encapsulated structure makes NSs more insensitive to external factors in optical and electrical properties. Furthermore, based on the internal open topology, NSs hybridized with a variety of functional materials have been fabricated, which is expected to confer TMD NSs with additional properties and functions attractive for potential application.

## Abstract (web summary)

Nanoscrolls (NSs) have interesting properties due to papyrus-like rolled-up geometry but their synthesis is difficult. Here Cui et al. show that vapor deposited monolayer transition metal dichalcogenide flakes form patternable arrays of NSs with the assistance of one droplet of ethanol solution.

## Introduction

Atomically two-dimensional (2D) transition metal dichalcogenides (TMDs), such as MoS_2_, MoSe_2_, WS_2_, and WSe_2_, display numerous exceptional electronic and optical properties arising from quantum confinement^1,2^. Complementary to gapless graphene, 2D TMDs with intrinsic band gaps are promising interesting field-effect transistor (FET) and optoelectronic devices. Recently, the main focus of attention has been for the production of their intrinsic or heterojunction structures, their properties, and their applications on 2D scale^3–8^. In addition to changes in 2D size and form, self-assembly of the atomically thin TMD flakes, as an emerging area, is largely unexplored. Assembly processes by folding and scrolling, i.e., rolling up, can transform relatively simple structures into complex topologies, such as nanoscrolls (NSs), with distinct properties as well as the original excellent characteristics. Indeed, theoretical calculations have predicted the unique topology of 2D material-based NSs to yield unusual electronic and optical properties^9–12^; thus these NSs have promise as building blocks in flexible electronics, microfluidics, energy storage, self-propelled micromachines, and optical resonators^13–18^. However, experimental realization of such high-quality NSs has only been achieved for graphene^13–16^ and boron nitride^19^, which exhibited high strength and chemical inertness. Limitations in mechanical strength and chemical stability present difficulties in producing high-quality TMD-NSs^20^. Recently, an argon plasma-assisted method has been demonstrated for the fabrication of amorphous MoS_2_-NSs, but in this method MoS_2_ sheets failed to scroll effectively, only edge region of the sheet curved while the large central area remains plane. Moreover, upon argon plasma etching, a serious damage has been brought to the original sheets, where almost half of sulfur atoms were removed. The obtained NSs exhibited low crystallinity even to be amorphous^21^. A high-quality TMD-NS is highly desirable for both fundamental studies and potential applications; however, its reliable experimental fabrication still remains challenging.

Here, we show a very simple method for fabricating high-quality TMD-NSs, which only requires one droplet of ethanol solution to scroll chemical vapor deposition (CVD)-grown monolayer TMD flakes in situ in 5 s with a nearly 100% yield. TMD-NSs are promising optoelectronic materials with potential applications in optoelectronic devices because of the high FET mobility. Owing to self-encapsulated structure, the optical and electrical properties of NSs are more insensitive to external factors, while the electrical performance of 2D materials flakes varies with both the underlying substrate and the environment. In addition, because of the internal open topology, the interlayer spacing of TMD-NSs can be easily expanded to accommodate a variety of functional materials, including organic small molecules, polymers, nanoparticles, and 2D materials, as well as biological substances. These features are very attractive for applications in solar cells, photodetectors, flexible logic circuits, energy storage, and sensors.

## Results

### Rolling up CVD-grown TMD monolayer flakes

**Fig. 1:**
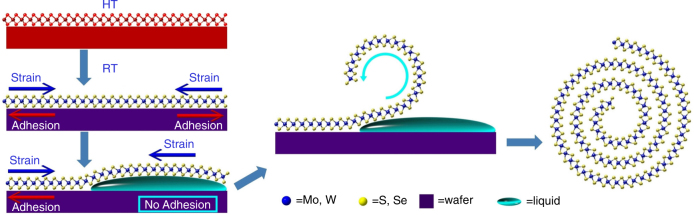
Schematic of self-scrolling of chemical vapor deposition (CVD)-based transition metal dichalcogenide (TMD) monolayer flakes. A proposed series of steps leading to the formation of TMD-NSs is shown: (I) initial TMD monolayer growth on a substrate at a high temperature (HT) (e.g., 720, 825 °C); (II) the generation of a strain, balanced with the adhesion from substrate, in the TMD monolayer while cooling down to room temperature (RT); (III) the insertion of liquid between the TMD monolayer and substrate and the disappearance of the adhesion; (IV) the TMD flakes curve out of the plane driven by the strain; (V) the final formation of TMD-NSs

In a typical experiment, large-area, monolayer TMD flakes were synthesized on a SiO_2_/Si substrate by CVD. Then, the TMD flakes were scrolled into NSs in just 5 s with a nearly 100% yield by placing one drop of ethanol solution (volume ratio of ethanol:water = 2:1) on their surface. Other aqueous solutions, such as methanol, tetrahydrofuran, dimethylformamide, and *N*-methyl-2-pyrrolidone, can also be used for TMD flakes scrolling (Supplementary Fig. 1, 2 and Supplementary Note 1). In addition, the scrolling proceeded well for TMD flakes grown on other substrates, such as Si_3_N_4_ and sapphire (Supplementary Fig. 3). To clarify the TMD-NSs formation process, the entire procedure was recorded using a CCD camera equipped on an optical-microscope (Supplementary Movie 1). A series of steps that might have led to the formation of the TMD-NSs are proposed in Fig. 1. Monolayer TMD flakes were grown on a substrate at a high temperature ( ≥ 720 °C). During the process of cooling down to room temperature, a strain was introduced to the TMD flakes because of the mismatch in thermal expansion coefficients between the TMD flake and the substrate. Stable TMD flakes were achieved when the strain and substrate adhesion were balanced. When an ethanol solution spread onto the surface of TMD flakes, the liquid film intercalated into the TMD flakes and the substrate, as demonstrated by the contrast color change in the flakes (Supplementary Fig. 4). With the liquid intercalation, part of the TMD flake was first released from the substrate to become freestanding as shown in Fig. 1. Under the built-in strain, the released part of the flake curved out of the plane and continued to roll up to a complete NS in the solution. An energy analysis of the MoS_2_ flakes before and after scrolling was presented in the Supplementary Note 2. The energy barrier required to bend the freestanding flake out of plane was proved to be relatively low, indicating the easy nucleation of a scroll. In addition, the strain energy induced from the thermal mismatch of the MoS_2_ flake was found high enough to activate the formation of scrolls after the liquid intercalation and the adhesion energy of the MoS_2_ flake with substrate decreasing (Supplementary Fig. 5). Strain as the stimulus to the NSs formation was also suggested by the following fact. When treated with ethanol solution, freshly produced TMD flakes on substrates were more prone to scroll in the shortest time with the highest yield, while the aged samples exhibited slightly decreased scrolling speed and yield. A few cracks were observed in aged samples by scanning electron microscopy (SEM, Supplementary Fig. 6), and these cracks could have partially relieved the strain.

**Fig. 2:**
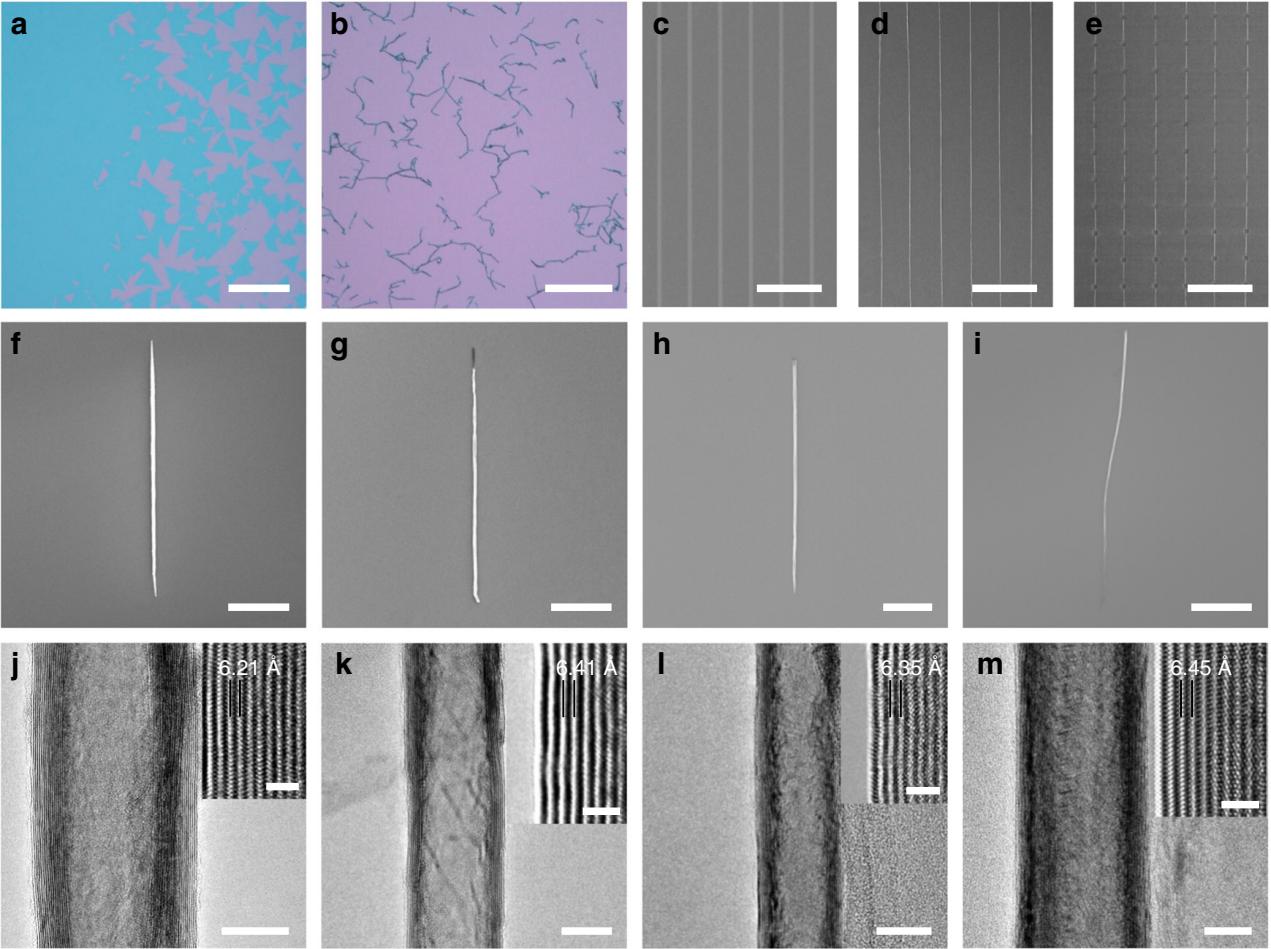
Transition metal dichalcogenide (TMD)-nanoscrolls (NSs) from self-scrolling chemical vapor deposition (CVD)-based TMD monolayer flakes. **a** Optical image of CVD-grown MoS_2_ monolayer flakes on a SiO_2_/Si substrate (the purple area represents the substrate, the green area represents the MoS_2_ monolayers; scale bar, 500 μm). **b** Optical image of MoS_2_-NSs on a SiO_2_/Si substrate (scale bar, 100 μm). **c**–**e** The fabrication process of a MoS_2_-NS array (scanning electron microscopy—SEM image shown, scale bars, 50 μm). **c** Large-area MoS_2_ monolayer film patterned into ribbons by focused ion beam (FIB) etching (the dark region represents MoS_2_ film, the bright area is bare substrate). **d** Long MoS_2_-NSs made from the controllable scrolling of the patterned MoS_2_ film in **c**. (The white parallel lines are MoS_2_-NSs, and the dark area represents the substrate). **e** A 12 × 6 array of MoS_2_-NSs fabricated via a second FIB etching of the long MoS_2_-NSs in **d**. **f**–**i** SEM images of typical TMD-NSs on SiO_2_/Si substrates: MoS_2_-NSs (**f**),WS_2_-NSs (**g**), MoSe_2_-NSs (**h**), and WSe_2_-NSs (**i**). (Scale bars, 5 μm in **f**, **i** and 10 μm in **g**, **h**). **j**–**m** TEM images of typical TMD-NSs: MoS_2_-NSs (**j**),WS_2_-NSs (**k**), MoSe_2_-NSs (**l**), and WSe_2_-NSs (**m**). (Scale bars, 20 nm). Inset: High-magnification images of sidewalls of TMD-NSs (scale bars, 2 nm)

TMD flakes were observed to prefer to scroll along one edge where the adhesion with the substrate was first relaxed upon the intercalation of the liquid. As the edges of CVD-grown TMD single crystals have a zigzag orientation^4,22,23^, the chirality of their NSs are defined (Supplementary Fig. 7). For large-area polycrystalline films without regular edges, the orientation and chirality of their NSs are generally random (Fig. 2a, b). Thus, the controllable scrolling of TMD films was attempted (Fig. 2c–e and Supplementary Fig. 8). Using a focused ion beam (FIB), a MoS_2_ polycrystalline film was patterned into parallel ribbons with defined width and direction (Fig. 2c) to produce long NS arrays (Fig. 2d). Using FIB etching a second time, long NSs were further arranged into periodic arrays with a controlled length (Fig. 2e). These NS arrays have promise for wide application, such as integrated circuits and matrix displays. We assume that horizontal TMD-NS arrays with controllable chirality could potentially be achieved by controlling the crystal orientation of the ribbon edges via etching from one large-area TMD single crystal, which is prevented by the technique to synthesize large-area TMD single crystals.

### Morphology characterization

CVD-grown TMD flakes are high quality even comparable to intrinsic flakes^23,24^. The ethanol solution was clean and harmless; therefore, no defects or impurities were introduced into the high-quality TMD-NSs (Supplementary Fig. 9). Based on this method, several typical TMD-NSs, including those of MoS_2_ (Fig. 2f and Supplementary Fig. 10), WS_2_ (Fig. 2g), MoSe_2_ (Fig. 2h), and WSe_2_ (Fig. 2i), were successfully fabricated. Most of the NSs displayed a straight and compact appearance. High-resolution transmission electron microscopy (HR-TEM) was used to further examine the TMD-NSs microstructure, which was found to be multiwalled and tubular (Fig. 2j–m). The diameter and sidewall thickness of the TMD-NS varied with the size of the original flake. The hollow diameters were 10–40 nm. In addition, the high-magnification images of the sidewalls and the selected area electron diffraction (SAED) both showed that the TMD layers in the NSs were stacked uniformly and compactly (Fig. 2j–m inset and Supplementary Fig. 11). The distance between adjacent walls was approximately 6.21 Å, 6.41 Å, 6.35 Å, and 6.45 Å for MoS_2_-, WS_2_-, MoSe_2_-, and WSe_2_-NSs, respectively, which matched the interlayer spacing in bulk TMDs (6.15 Å, 6.36 Å, 6.45 Å, and 6.49 Å for MoS_2_, WS_2_, MoSe_2_, and WSe_2_, respectively), indicating both the tight scrolling of the TMD flakes and the lack of interlayer contamination.

### Optical characterization

**Fig. 3:**
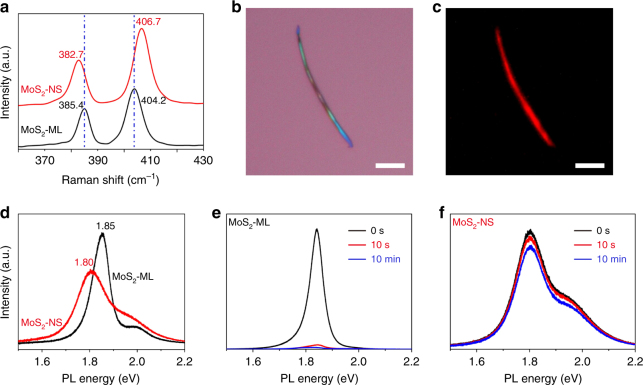
Optical characterization of MoS_2_-NSs (nanoscrolls). **a** Raman spectra of chemical vapor deposition (CVD)-grown MoS_2_ monolayers (MoS_2_-ML, black line) and MoS_2_-NSs (red line) with 532 nm excitation. **b** Optical image of a typical MoS_2_-NS. **c** Corresponding fluorescence image of the MoS_2_-NS in **b**. The fluorescence image was obtained with an excitation wavelength of 510–560 nm. **d** PL spectra from MoS_2_ monolayer (black line) and MoS_2_–NSs (red line) (532 nm excitation). **e**, **f** PL spectra response of MoS_2_ monolayer (**e**) and MoS_2_-NSs (**f**) with NH_3_ doping for 0 s (black line), 10 s (red line), and 10 min (blue line) (scale bars, 5 μm in **b** and **c**)

Raman spectra of the TMD-NSs were recorded using 532 nm excitation (Fig. 3a and Supplementary Fig. 12a–c). The Raman signature of monolayer MoS_2_ flakes is consistent with that of mechanically exfoliated monolayer MoS_2_^25^. A similar Raman signature was also found for MoS_2_-NSs, demonstrating the good crystallinity of the MoS_2_-NSs. A blue shift was observed in the A_1g_ mode from 404.2 cm^−1^ for the monolayer MoS_2_ to 406.7 cm^−1^ for the MoS_2_-NSs, and this shift was attributed to the van der Waals interactions between neighboring NS layers^25,26^. Additionally, a red shift in the E^1^_2g_ mode was detected from 385.4 cm^−1^ for the monolayer MoS_2_ to 382.7 cm^−1^ for the MoS_2_-NSs, resulting from stacking-induced structural changes, long-range Coulombic interlayer interactions^25,27^, or/and a slight tension owing to the bending deformation of the flake^28^. The frequency shift behavior reflects structural changes after scrolling, and the changes were also observed for WS_2_-, MoSe_2_-, and WSe_2_-NSs (Supplementary Fig. 12a–c).

Semiconducting TMD monolayers are direct-bandgap materials because of quantum confinement effects^29^. The strong photoluminescence (PL) and a wide range of band gaps of the monolayer TMD semiconductors indicate their potential for use in 2D optoelectronics, such as optical energy conversion devices^30^or photodetectors^31^. Despite having a stacking structure similar to that of bulk TMDs, these TMD-NSs were shown to have an obvious PL, in contrast with the bulk counterparts, in which PL is negligible^29^. Red luminescence from a typical MoS_2_-NS was clearly observed by fluorescence imaging, indicating that the PL was sufficiently strong to be imaged (Fig. 3b, c). In the PL spectra of Fig. 3d, A, B direct excitonic transitions^29^ from MoS_2_-NSs appeared at around 1.80 eV (688 nm) and 1.95 eV (635 nm), matching their absorption resonances (Supplementary Fig. 13). Compared with monolayer MoS_2_, a red shift in the A exciton transition, approximately of 30–60 meV was found for MoS_2_-NSs and was accompanied by an increase in the full-width at half-maximum of 50–80 meV. The red shift of A peak, an indicator of the variation of the electronic structure, can be explained by the stacking and tension effects in the NSs^29,32,33^, while PL peak broadening is presumably owing to the overlap of signals from several MoS_2_ layers in a NS. Analogous PL behavior was also observed for the WS_2_-, MoSe_2_-, and WSe_2_-NSs (Supplementary Fig. 12d–f).

It is well known that monolayer TMD atoms are fully exposed, which makes their optical and electrical properties sensitive to environmental factors. As shown in Fig. 3e, f, when exposed to NH_3,_ the PL intensity of monolayer MoS_2_ flakes was quenched rapidly and drastically; in contrast, the PL intensity of MoS_2_-NSs did not show a pronounced decrease. Apparently, the optical properties of MoS_2_-NSs were less dependent on the environment, which might be attributed to the self-encapsulated structure, where most of the MoS_2_ flakes were rolled up inside and sealed by the outermost layers.

### FET performance

**Fig. 4:**
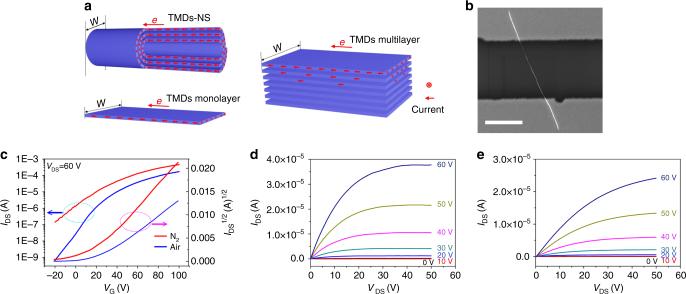
Electrical characteristics of MoS_2_-NSs (nanoscrolls). **a** Schematic representations of current conduction in transition metal dichalcogenide (TMD)-nanoscrolls (NSs), TMDs monolayer, and TMDs multilayer under bias. The *W* represents the conduction channel width. The NSs made from the below TMDs monolayer have a much shorter *W*. For TMDs multilayer, the current only passes through the outermost shell layers. **b** SEM image of a typical MoS_2_-NS FET (scale bar, 10 μm). **c** Transfer characteristics of MoS_2_-NS tested in N_2_ (red) and air (blue). **d** Output characteristics of MoS_2_-NS tested in N_2_. **e** Output characteristics of MoS_2_-NS tested in air

Since TMD-NSs are semiconductors, and there are no previous reports on their FET properties, it is interesting to explore this issue here. Device characterization is easy for MoS_2_-NSs because the NSs have been produced on a SiO_2_/Si substrate. Defining source and drain electrodes were directly deposited on the top of MoS_2_-NSs via a shadow mask. This process avoided organic impurity contamination that might occur during traditional electron-beam lithography. The FETs based on the MoS_2_-NSs exhibited good FET behavior, and the current/voltage characteristics conformed well to conventional transistor models in both saturated and linear regimes in N_2_ (Fig. 4 and Supplementary Fig. 14). According to the equation below, we calculated the mobility in the saturation regime.

$$I_{\mathrm{D}} = \left( {W/2L} \right)C_{\mathrm{i}}\mu \left( {V_{\mathrm{G}} - V_{\mathrm{T}}} \right)^2,$$

where *L* and *W* are the channel length and width, respectively, *C*_i_ is the insulator capacitance per unit area (11 nF cm^−2^), *µ* is the FET mobility, and *V*_T_ is the extrapolated threshold voltage. More than 300 devices were tested. The electron mobility of NSs was measured to be mostly in the range 200–700 cm^2^ V^−1^ s^−1^, with an on/off ratio of over 1.0 × 10^5^. The mobility of MoS_2_-NSs, nearly 30-fold greater than that of monolayer MoS_2_ flakes (10–20 cm^2^ V^−1^ s^−1^)^34^, should be ascribed to the high current density for compact scroll topology. Invoking an Archimedean spiral model, we quantified the change in the conduction channel widths upon scroll (the detailed analysis is provided in Supplementary Note 3). A considerable reduction in conduction channel widths was achieved after scrolling, while there is no significant decline in the current for the flakes upon scrolling process (Supplementary Fig. 15, 16). This led to a higher current density and mobility. In addition, benefiting from the compact scrolling, conductive layers in a NS compactly stacked layer by layer. The adjacent layers acted as ideal substrates for each other with atomically smooth surface, relatively no dangling bonds and charge traps, which also enhanced electron transporting efficiency^35^. With the similar stacked layer structures, however, the MoS_2_-NSs also exhibited much greater electron mobility than did the multilayer MoS_2_ flakes^36^. It can be explained that in a MoS_2_-NS, electrons are transported through the whole scrolled MoS_2_ flake, while in multilayer MoS_2_ flakes, electrons are blocked by van der Waals gaps while migrating to inner layers; thus, only a few shell layers contribute to the carriers transmission (Fig. 4a). The monolayer MoS_2_-based FETs were observed to be very sensitive to atmosphere. However, there was no obvious decrease in FET mobility for the MoS_2_-NSs when they were tested in air (Fig. 4c–e) because most of each electrically active MoS_2_ layers is encapsulated within each NS and the compact stacked (with an interlayer distance of 0.62 nm) morphology can efficiently prevent oxygen from intercalating into the MoS_2_-NSs to deteriorate transistors performance. The high mobility and unique self-encapsulated morphology make MoS_2_-NSs promising in FETs.

### Hybrid TMD-NSs

**Fig. 5:**
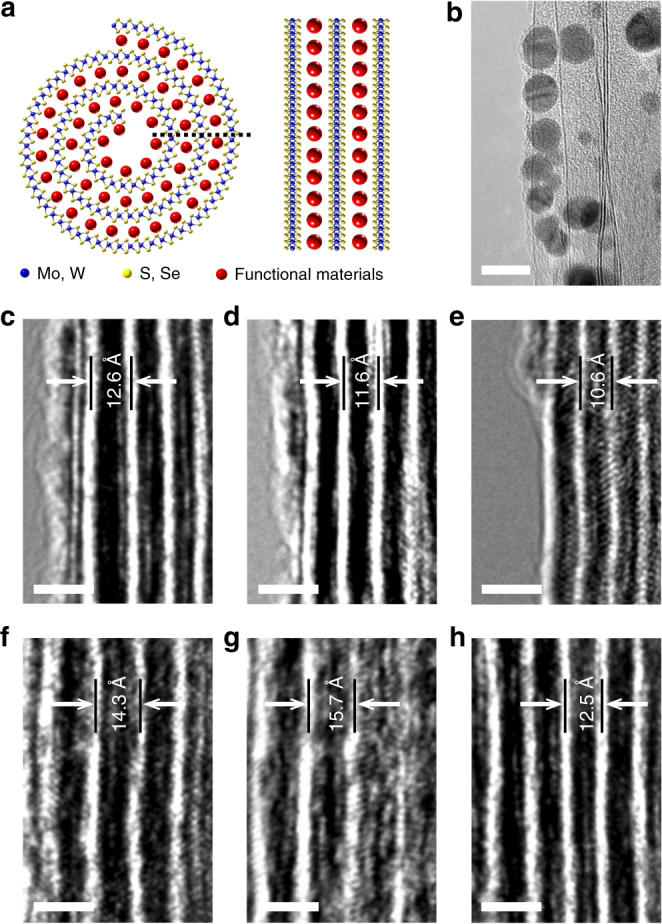
Hybrid transition metal dichalcogenide (TMD)-nanoscrolls (NSs). **a** Schematic showing the cross section and sidewalls of hybrid TMD-NSs. **b**–**h** HR-TEM images of sidewalls of MoS_2_-NSs hybridized with AuNPs (**b**), GO (**c**), pentacene (**d**), CuPc (**e**), PDPP3T (**f**), DNA (**g**) and polypeptide (**h**). AuNPs approximately 5 nm in diameter were clearly observed between the walls; the GO flakes were also distinguishable, as shown in **c**. Lattice expansion was displayed in all hybrid NSs above. The bright white walls represent the MoS_2_ layers in (**c**–**h**) (scale bars, 10 nm in **b** and 2 nm in **c**–**h**)

As mentioned above, TMD-NSs are inert to the outsides owing to their self-encapsulated structures; however, they are inclined to be doped and form composites because of their internal open topological structures. With a pretreatment, TMD flakes can be hybridized with diverse loads, which become sandwiched into the van der Waals gaps upon flakes scrolling (Fig. 5a). In this paper, we produced MoS_2_-NSs hybridized with gold nanoparticles (AuNPs) (Fig. 5b), graphene oxide (GO) (Fig. 5c), pentacene (Fig. 5d), copper (II) phthalocyanine (CuPc) (Fig. 5e), poly{2,2ʹ-[(2,5-*bis*(2-hexyldecyl) −3,6-dioxo- −2,3,5,6-tetrahydropyrrolo[3,4-c]pyrrole-1,4-diyl)dithiophene] −5,5ʹ-diyl-alt-thiophen- −2,5-diyl} (PDPP3T) (Fig. 5f), DNA (Fig. 5g) and polypeptide (Fig. 5h). In contrast with nanotubes, the lattice spacing of NSs can adjust automatically upon hybridization with foreign substances of different sizes, and this adjustment can be evaluated by HR-TEM. Figure 5b–h displayed the lattice spacing of hybrid NSs, where lattice expansion was observed for all composite NSs, and some composite components, such as AuNPs, were large enough to be directly visualized (Fig. 5b). Moreover, Raman spectroscopy was utilized to verify the presence of foreign substances. As a typical example, the intercalation of GO into NSs was verified by the emergence of the D, G, and 2D peaks of GO (around 1350, 1600, 2671 cm^−1^)^37^, as well as vibrational modes (around 383, 406 cm^−1^) for MoS_2_ (Supplementary Fig. 17a). In addition, TMD-NSs can be conferred with additional properties and functions upon hybridization with other functional materials, such as organic small molecules, polymers, nanoparticles, and 2D materials, as well as biological substances. For example, after hybridization with PDPP3T, a new fluorescence peak appeared at about 1.42 eV (873 nm) for MoS_2_-NSs (Supplementary Fig. 17b), indicating that the hybridization broadened MoS_2_-NS emission spectrum and could eventually extend their optical applications. Furthermore, the intercalated NS by PDPP3T exhibited a good stability in air, due to the self-encapsulated structure (Supplementary Fig. 18).

## Discussion

In summary, we have explored a simple method for producing high-quality, tightly scrolled TMD-NSs from CVD-grown monolayer TMD flakes with the assistance of one drop of an ethanol solution. An obvious PL was shown in TMD-NSs, with a red shift in the direct excitonic transitions. The electron mobility of the MoS_2_-NSs was approximately 30-fold greater than that of the monolayer MoS_2_ flakes. This scrolling strategy will be a candidate for increasing mobility of other 2D materials. Owing to their unique self-encapsulated structures, these TMD-NSs show stable optical and electronic properties in different atmospheres. The HR-TEM results show the internal open topological structure of the TMD-NSs, which serve as effective carriers for accommodating external substance of different sizes in their tunable van der Waals gaps. The hybridization with other functional materials will confer TMD-NSs with additional properties and functions attractive for potential application.

## Methods

### CVD growth of TMD monolayers

TMD monolayers were grown by an atmospheric pressure CVD. The growth substrates, including SiO_2_/Si, Si_3_N_4_ and sapphire, were cleaned in acetone, isopropanol, and deionized water and then dried under high-purity nitrogen flow.

For MoS_2_ monolayers, the growth substrate was placed face-down above a ceramic boat containing MoO_3_ powder (Sigma Aldrich, 99.998%, 20 mg). The ceramic boat was then loaded in the heating zone center of the furnace tube, where another boat containing 80 mg of sulfur (Alfa Aesar, 99.999%) was located upstream. The tube was first purged with ultrahigh-purity argon for 10 min at a flow rate of 200 sccm. Then, the furnace was heated from room temperature to 500 °C in 12 min, 500–720 °C in another 20 min, and then stayed at 720 °C for 5 min. The sulfur was heated to 130 °C with a separate heat setup as the furnace reached 720 °C. Finally, the temperature was cooled from 720 to 570 °C in 20 min before opening the furnace for rapid cooling. Argon as carrier gas was maintained at a flow rate of 10 sccm throughout the growth process.

The growth of WS_2_ monolayers was similar to that of MoS_2_, except that WO_3_ (Alfa Aesar, 99.998%, 10 mg) was used as one of the precursors and the highest growth temperature was set at 825 °C, with sulfur at 150 °C.

WSe_2_ monolayers were grown on substrates with WO_3_ (10 mg) and selenium powders (Alfa Aesar, 99.999 + %, 1–5 mm) as precursors. The growth recipe was as follows: ramp the furnace from room temperature to 825 °C in 20 min, sit 20 min at 825 °C, cool to 600 °C in 20 min, and open the furnace for rapid cooling. The selenium powders were heated to 300 °C when the furnace temperature rose to 825 °C and were maintained at this temperature for 40 min. 1.5 sccm hydrogen and 23.5 sccm argon were used during the growth process.

MoSe_2_ monolayers were grown via a procedure similar to that used for WSe_2_ monolayers, except that the source precursors were MoO_3_ and selenium, and the selenium was kept at 400 °C during the growth process.

### Fabrication of TMD-NSs

TMD-NSs were fabricated by dropping an ethanol aqueous solution onto the CVD-grown TMD monolayers and allowing it to dry naturally. Rod-like NSs were then obtained on the substrate. The concentration of the ethanol aqueous solution ranged from 5:1 to 1:1 (volume ratio of ethanol: water), with an optimum ratio of 2:1.

### MoS_2_-NSs arrays

A MoS_2_ polycrystalline film was first patterned into parallel ribbons with defined width and direction using a FIB. A drop of pure ethanol was dropped on the patterned film, and then a coverslip was put on top of it. A thin liquid layer of pure ethanol can be formed between the film and the coverslip. An ethanol solution (1:1) was then dropped on the edge of the coverslip. Drove by the concentration gradient, the water in the solution will slowly infiltrate into the pure ethanol under the coverslip in the gradient direction (as shown in Supplementary Fig. 8). The diffusion rate can be adjusted by the concentration of the ethanol solution. In this way one side of a ribbon was first controlled to scroll to produce a long NS. The long NS array was then fabricated and further arranged into periodic arrays with a controlled length by FIB etching a second time.

### Hybrid MoS_2_-NSs fabrication

Pentacene/MoS_2_-NSs: a 1-nm-thick layer of pentacene was deposited on the MoS_2_ monolayers on a SiO_2_/Si substrate by vacuum thermal evaporation, followed by immersion into a modulated ethanol solution (5:1). After 10 min, the substrate was picked up and allowed to dry naturally. The pentacene/MoS_2_-NSs were obtained. The CuPc/MoS_2_-NSs were prepared in the same way.

PDPP3T/MoS_2_-NSs: A PDPP3T solution was prepared by dissolving 2 mg of PDPP3T powder in 1 ml of toluene. After stirring overnight, the PDPP3T solution was spin-coated onto the MoS_2_ monolayers at a speed of 5000 rpm. After annealing at 70 °C for 20 min, the PDPP3T-coated MoS_2_ monolayers were then dipped into an ethanol solution (5:1). After 10 min, the MoS_2_ monolayers were picked up and dried naturally.

GO/MoS_2_-NSs: This procedure is similar to that used for PDPP3T/MoS_2_-NSs. Freshly exfoliated GO sheets were dispersed in *n*-hexane and then spin-coated onto MoS_2_ monolayers. After dropping an ethanol solution (2:1) onto the samples, the GO/MoS_2_-NSs were obtained.

Au/MoS_2_-NSs: MoS_2_ monolayers were dipped into an ethanol solution (2:1) containing AuNPs. Ten minutes later, the MoS_2_ monolayers were picked up and dried naturally. Similar procedures were used for producing both DNA/MoS_2_- and polypetide/MoS_2_-NSs.

### MoS_2_-NS-based FET

Freshly made monolayer MoS_2_ flakes on a SiO_2_/Si substrate were kept in nitrogen for 48 h. Then the MoS_2_ flakes were scrolled into NSs with one drop of ethanol solution (2:1) in nitrogen atmosphere. Bottom-gated transistors based on these MoS_2_-NSs were fabricated on the SiO_2_/Si substrate without additional transfer. 100 nm of Au was deposited on the top of MoS_2_-NSs as source and drain electrodes with a shadow mask by vacuum thermal evaporation at a rate of about 0.3 Å s^−1^. The electrical measurements were conducted under a nitrogen atmosphere and ambient condition, respectively.

### Characterization

Raman and PL measurements were performed with a 532 nm laser under ambient conditions (inVia-Reflex). All optical images and the video were captured with a Nikon Eclipse LV100D. The fluorescence microscope characterization was performed using a fluorescence microscope equipped with a mercury lamp as the excitation light source. Large-area MoS_2_ films and long MoS_2_-NSs were etched via FIB-SEM (FEI, Helios Nanolab G3 CX). SEM (Hitachi S-4800) and HR-TEM (JEOL JEM-2100F) were employed to image the samples. Current–voltage curves were obtained by a three-probe station under a nitrogen atmosphere and ambient conditions (Keithley 4200 SCS).

### Data availability

The data that support the findings of this study are available from the corresponding author on reasonable request.

## Electronic supplementary material

Supplementary Information(PDF 1454 kb)

Description of Additional Supplementary Files(DOCX 14 kb)

Supplementary Movie 1
