# Supplementary material for: Rolling up transition metal dichalcogenide nanoscrolls via one drop of ethanol
Source: Nat Commun. 2018 Apr 3;9:1301. doi: 10.1038/s41467-018-03752-5 (PMC5883047; doi:10.1038/s41467-018-03752-5)
Supplement: Supplementary file 1 — Supplementary Information(PDF 1454 kb) [file 41467_2018_3752_MOESM1_ESM.pdf]

Supplementary Information

Rolling up Transition Metal Dichalcogenide Nanoscrolls via One Drop of Ethanol

Xueping Cui, Zhizhi Kong, Enlai Gao, Dazhen Huang, Yang Hao, Hongguang Shen, Chong-an Di, Zhiping Xu, Jian Zheng*, Daoben Zhu

Supplementary Figures

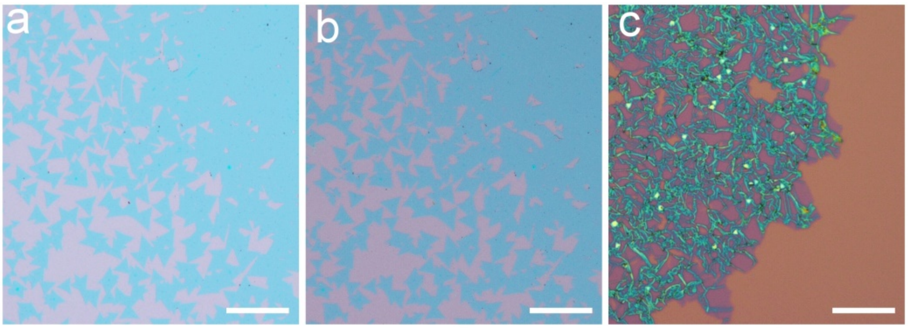

Supplementary Figure 1. a, Optical image of built-in tension released monolayer MoS_2 after transferred from growth substrate to another bare substrate. **b**, Optical image of the transferred sample after immersing into the ethanol solution. Almost no scroll was observed in the built-in tension released sample. **c**, Optical image of the MoS_2 sample after sharp intercalation of pure water. Loosely curled and disordered nanoscrolls with large unscrolled film were observed. (Scale bars, 200 μm in **a**, **b** and 20 μm in **c**).

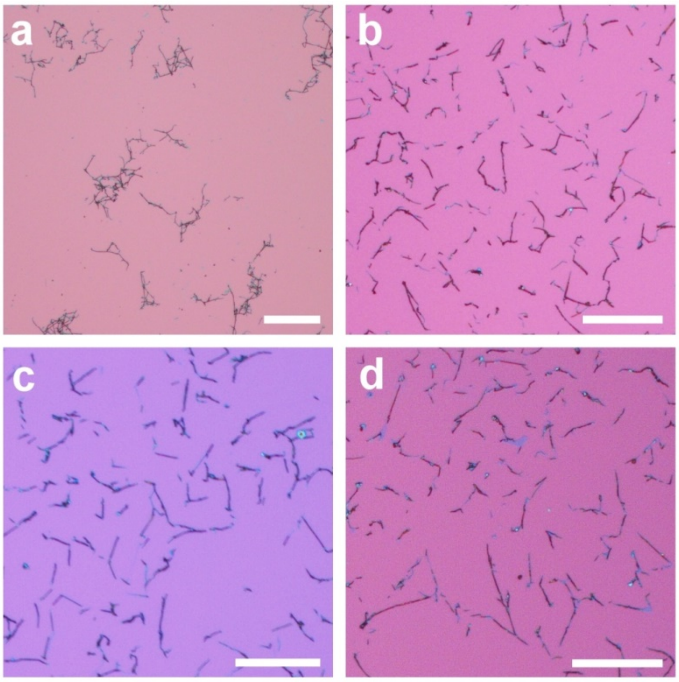

Supplementary Figure 2. MoS_2-NSs made in the aqueous solution of methanol (**a**), tetrahydrofuran (**b**), dimethylformamide (**c**), and N-methyl-2-pyrrolidone (**d**). (Scale bars, 100 μm in **a** and 50 μm in **b**-**d**). Tight, straight scrolling with high yield can also be obtained with different solutions at the optimized ratio.

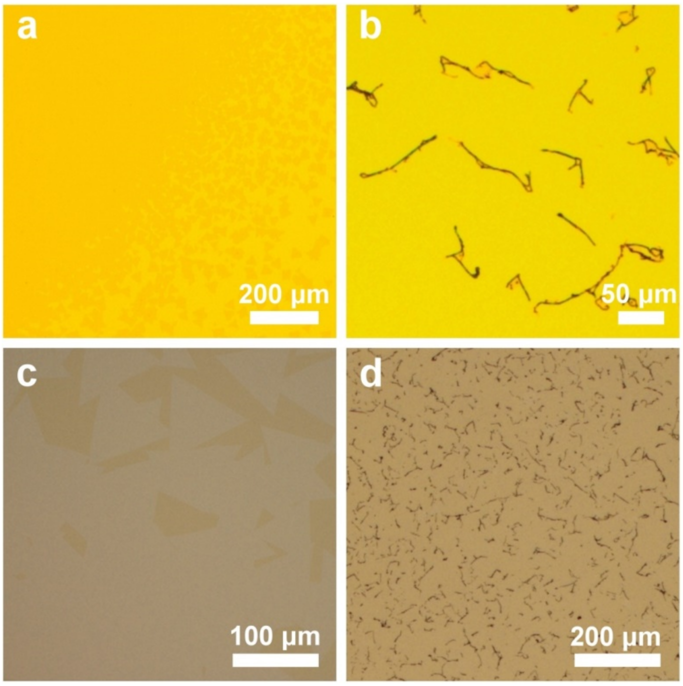

Supplementary Figure 3. MoS_2-NSs made from monolayer MoS_2 flakes grown on non-silicon substrates. **a**, Optical image of MoS_2 monolayers grown on a Si_3N_4 substrate. The dark yellow represents MoS_2 monolayers, and the light yellow is the Si_3N_4 substrate. **b**, Optical image of MoS_2-NSs made from MoS_2 monolayers grown on the Si_3N_4 substrate. **c**, Optical image of MoS_2 monolayers grown on a sapphire substrate. The white area represents MoS_2 monolayers, and the brown area represents the sapphire substrate. **d**, Optical image of MoS_2-NSs rolled up from MoS_2 monolayers grown on the sapphire substrate.

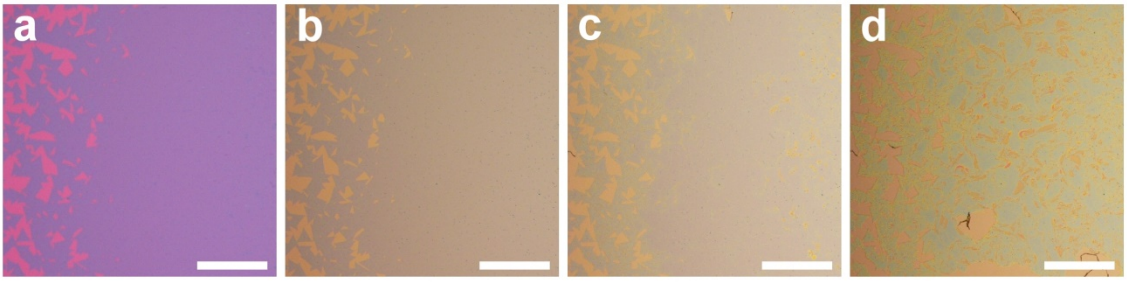

Supplementary Figure 4. The evolution in contrast color of MoS_2 flakes when treated by ethanol solution. a, Optical image of MoS_2 flakes on a SiO_2/Si substrate before dropping an ethanol solution. **b**, Optical image of MoS_2 flakes just covered with the ethanol solution. **c**, Optical image of MoS_2 flakes when a small amount of ethanol solution intercalating into MoS_2 flakes and the substrate **d**, Optical image of MoS_2 flakes when a large amount of ethanol solution intercalating into MoS_2 flakes and the substrate. (Scale bars, 100 μm).

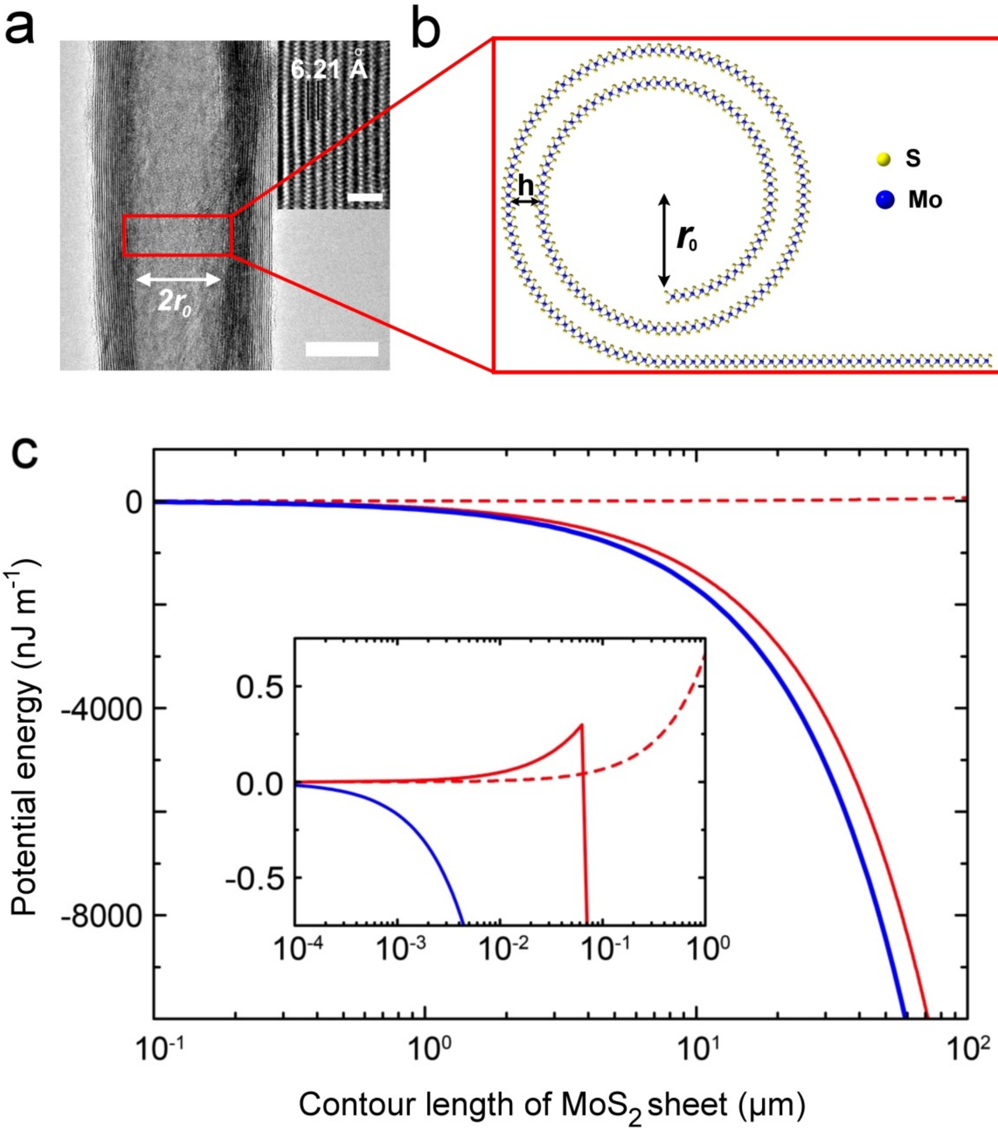

Supplementary Figure 5. **a**, The TEM image of a typical MoS_2-NS. **b**, a schematic illustration of the MoS_2-NS in (a). **c**, Potential energy analysis of a flat MoS_2 sheet (blue line), a flat MoS_2 sheet with solvent intercalation at its interface with the substrate (red dash line), and a scrolled MoS_2 sheet (red line), plotted as the function of length *l*.

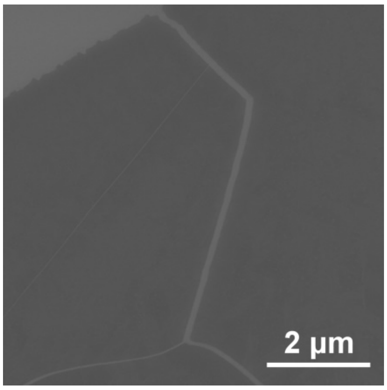

Supplementary Figure 6. SEM image of an aged MoS_2 sample on a SiO_2/Si substrate.

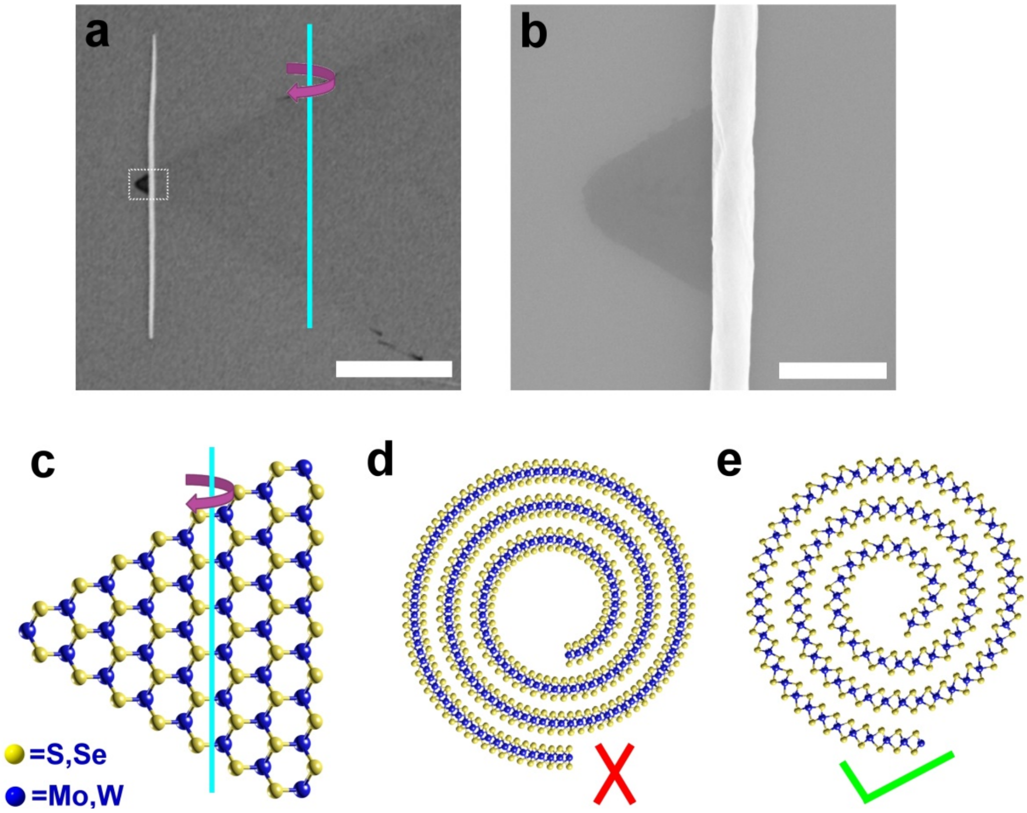

Supplementary Figure 7. Chirality of the monolayer MoS_2 single crystal scrolling. **a**, SEM image of a MoS_2-NS rolled up from a single-crystal MoS_2 monolayer. **b**, High-magnification SEM image of the rectangular area in (**a**). **c-e**, Schematic of scrolling chirality of TMD-NSs made from single-crystal flakes. The single-crystal TMDs flakes curled along the zigzag edge. (Scale bars, 10 μm in **a** and 500 nm in **b**)

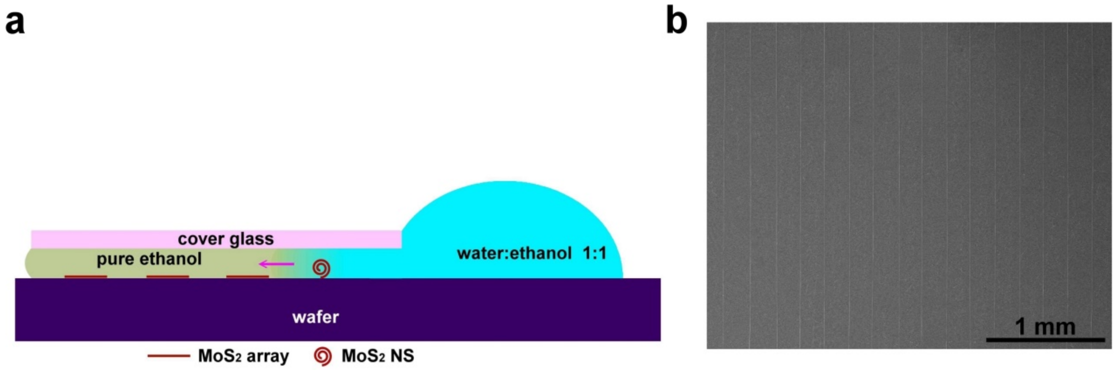

Supplementary Figure 8. a, Schematic of the preparation of a MoS_2-NS array. **b**, A long MoS_2-NS array with a maximum area of about 3 mm×3 mm on a SiO_2/Si substrate.

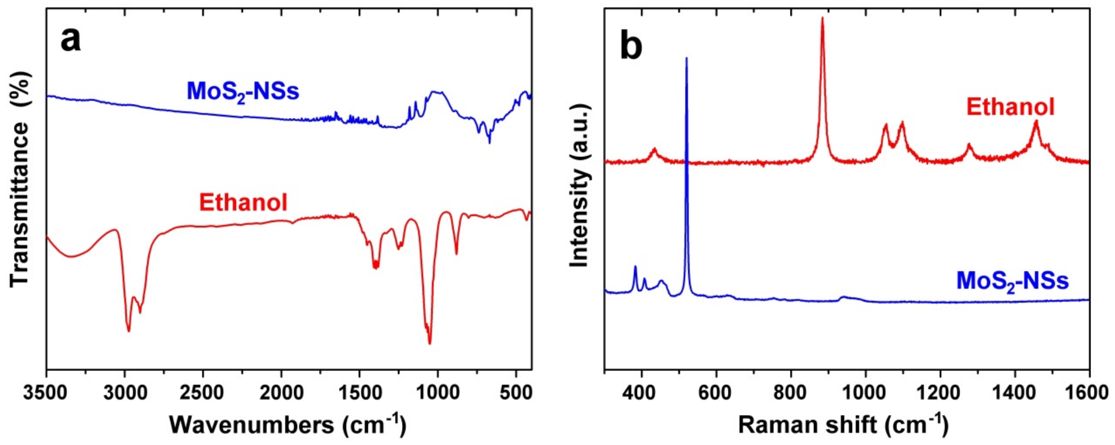

Supplementary Figure 9. Infrared (IR) and Raman analysis of MoS_2-NSs. a, IR spectrum of ethanol (red) and MoS_2-NSs (blue). **b**, Raman spectra of ethanol (red) and MoS_2-NSs (blue). A weak IR band at 481 cm^{-1} was assigned to Mo-S vibration. In the Raman spectra, vibration modes (at 382.9 cm^{-1}, 407.3 cm^{-1}) were clearly observed for MoS_2-NSs, without modes for ethanol in the NS. IR and Raman spectra both showed there was no residual ethanol in the nanoscrolls. The IR analysis was carried out under ambient conditions on a microscope setup (Nicolet iN10-iZ10). Raman characterization was performed with a 785 nm laser (inVia-Reflex).

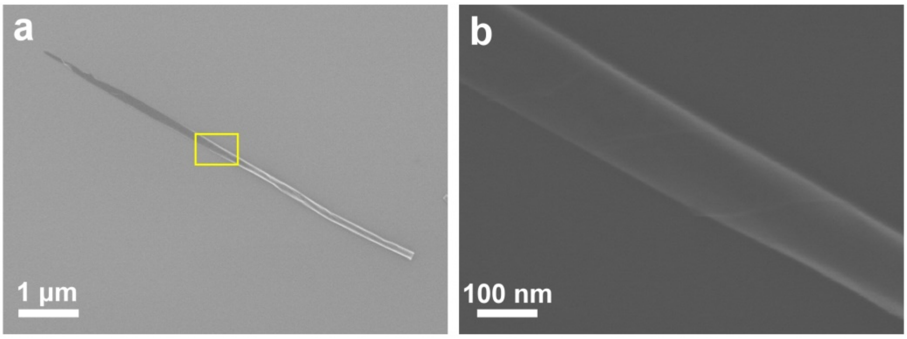

Supplementary Figure 10. a, SEM image of a MoS_2-NS. **b**, An enlarged image of the marked area in (a).

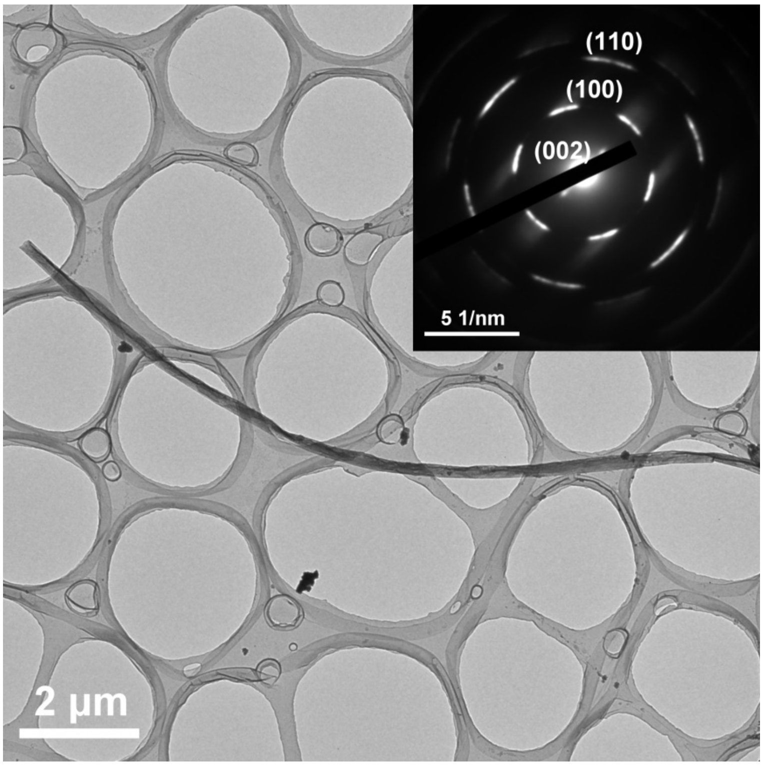

Supplementary Figure 11. TEM image and the selected-area diffraction (SAED) pattern (inset) of an individual MoS_2-NS. Two sharp diffraction spots, indexed to (002) plane, indicated the uniformity of the interlayer spacing in the NS. According to Bragg's diffraction equation, the interlayer spacing of the NS was about 6.18 Å, in good agreement with the HR-TEM observation.

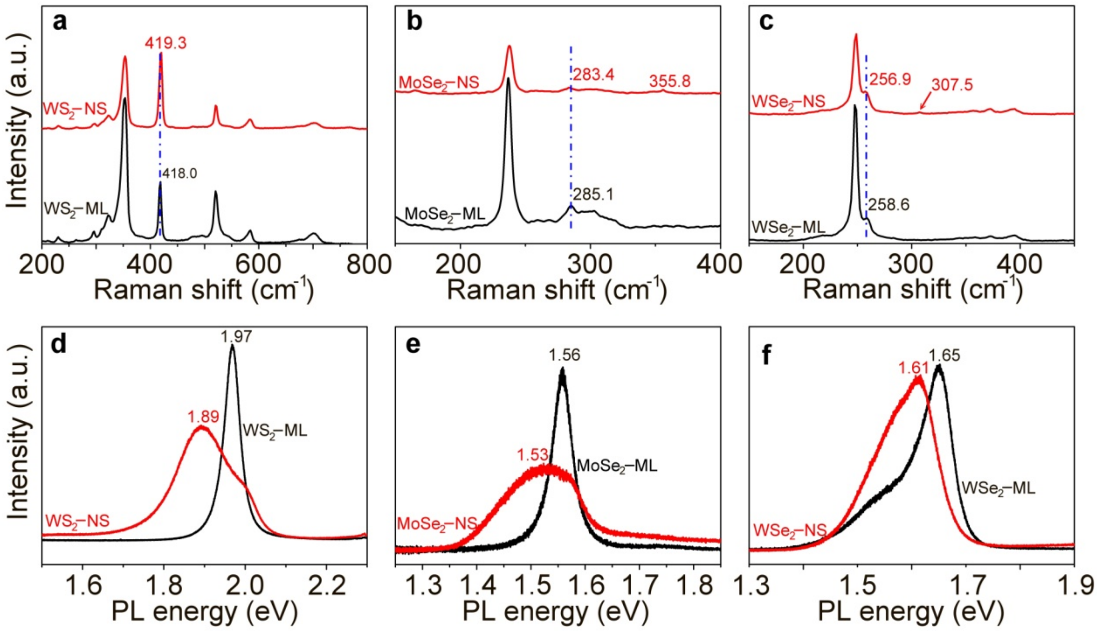

Supplementary Figure 12. Optical characterization of WS_2-, $MoSe_2$-, and WSe_2-NSs. a-c, Raman spectra of CVD-grown WS_2, $MoSe_2$, and WSe_2 monolayers (ML, black) and NSs (red), respectively. **d-f**, PL spectra of CVD-grown WS_2, $MoSe_2$, and WSe_2 monolayers (black) and NSs (red) (532 nm excitation).

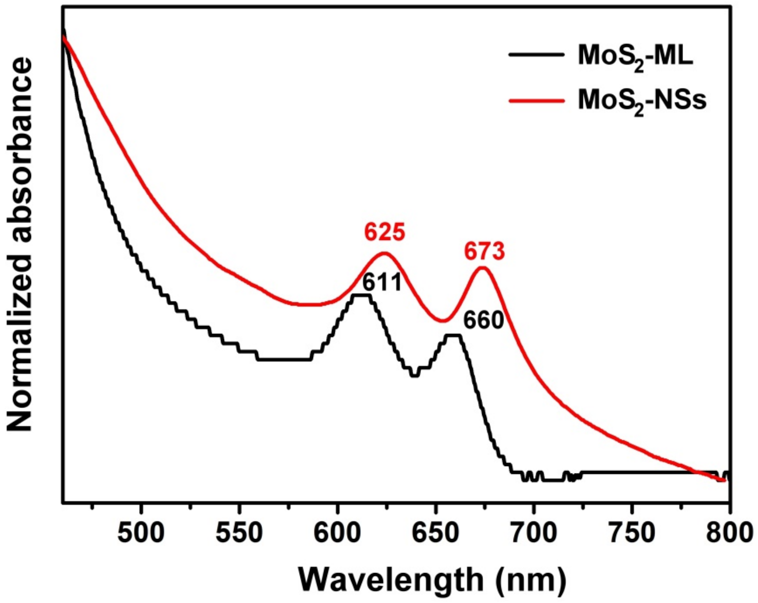

Supplementary Figure 13. Optical absorption characterization of MoS_2 monolayers (MoS_2-ML) and MoS_2-NSs. Monolayer MoS_2 and the thin film of NSs were both measured on a fused quartz substrate in an integrating sphere set-up (UV-2600). The NS film was prepared by the accumulation of the multiple transferred nanoscrolls from SiO_2/Si substrate and the spectra were normalized for better comparison. Obvious red shift in absorption resonances was observed after scrolling, corresponding to the red shift of exciton transitions in the PL spectra.

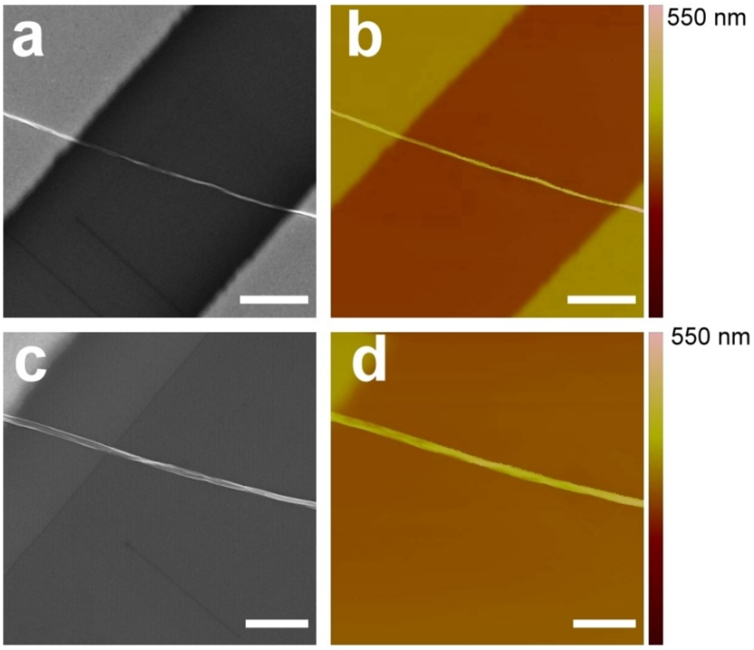

Supplementary Figure 14. High-resolution scanning electron microscope (HR-SEM) and atomic force microscope (AFM) characterization of MoS_2-NS FET in Figure 4b. a, HR-SEM image of the MoS_2-NS FET. **b**, AFM image of the MoS_2-NS FET showed in (a). **c**, HR-SEM image of part of the MoS_2-NS FET in (a). **d**, AFM image of the MoS_2-NS FET showed in (c). (Scale bars, 5 μm in **a**,**b** and 2μm in **c**,**d**)

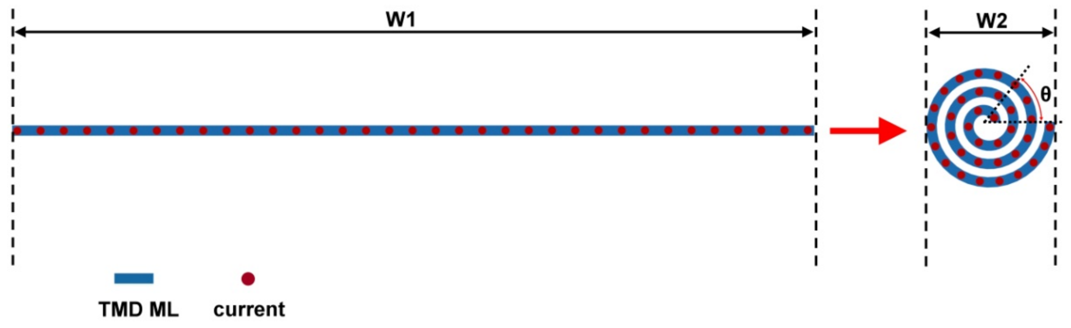

Supplementary Figure 15. The schematic of a monolayer TMD flake rolled into a NS along the conduction channel width direction. $W1$, $W2$ represent the conduction channel widths of TMD flake and the NS respectively. θ is the torsion angle expressed in polar coordinates.

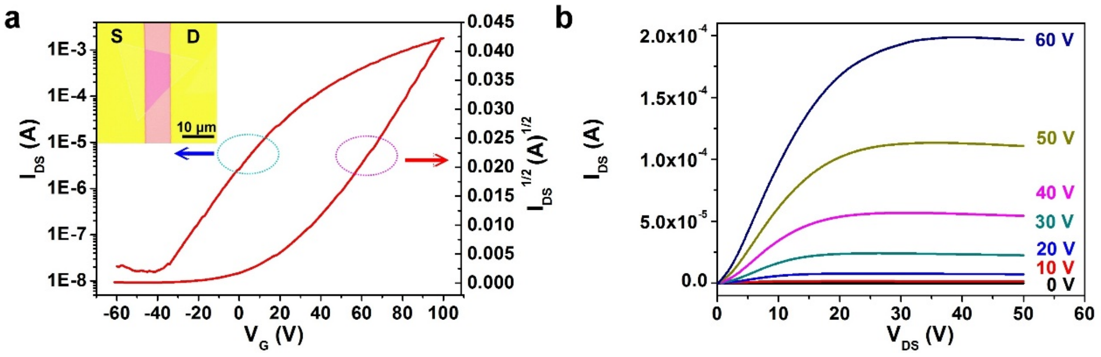

Supplementary Figure 16. Electrical characterizations of monolayer MoS_2 FETs. **a**, Transfer curve of a back-gated monolayer MoS_2 FET measured in N_2 at room temperature. The source-drain bias was fixed at 60 V. Inset: Photograph of the FET device made from a monolayer MoS_2 flake grown on a SiO_2/Si substrate by CVD. The monolayer MoS_2 flake has similar dimensions as the flake that rolled into the NS in Figure 4b. **b**, Output curve of the monolayer MoS_2 FET. Experimented results show that the flake FET has a similar saturated current to that of the NS FET under the same bias. There is no significant decline in saturated current for the flakes upon scrolling process.

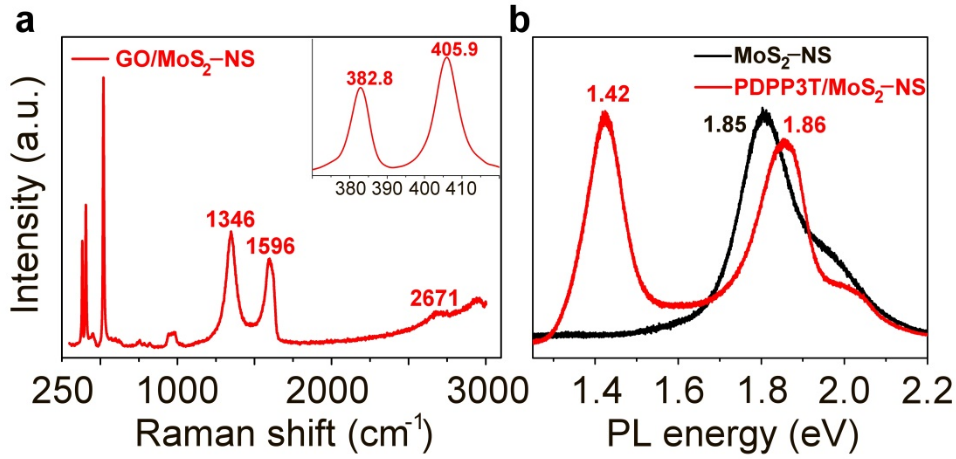

Supplementary Figure 17. Optical characterization of hybrid MoS_2-NSs. a, Raman spectrum of GO/MoS_2-NSs. The inset is a magnified view of the range of 370-420 cm^{-1}. **b**, PL spectra of MoS_2-NSs (black) and PDPP3T/MoS_2-NSs (red) (532 nm excitation).

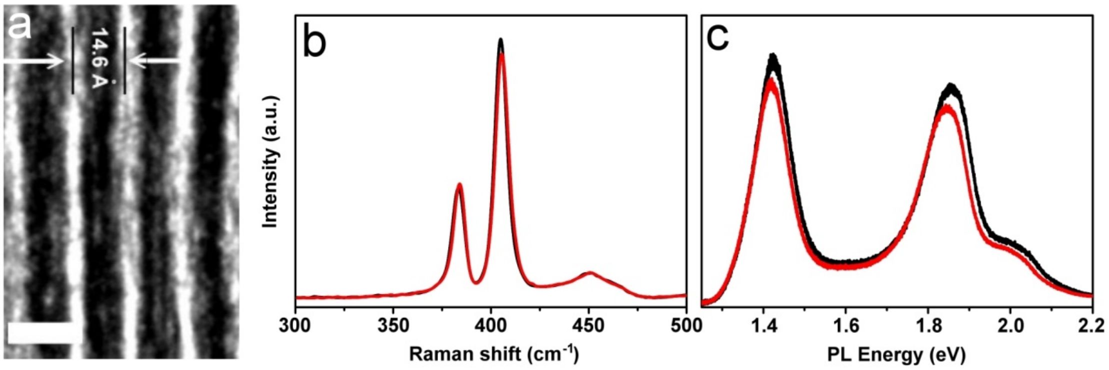

Supplementary Figure 18. a, HR-TEM images of PDPP3T/MoS_2-NSs one month after the first intercalation. **b**, Raman spectra of PDPP3T/MoS_2-NSs placed in ambient condition for 0 day (black line) and one month (red line). **c**, PL spectra of PDPP3T/MoS_2-NSs placed in ambient condition for 0 day (black line) and one month (red line). (532 nm excitation)

Supplementary Notes

Supplementary Note 1. The role of immersing solution in CVD-based TMD flake scrolling

To clarify the role of the immersing solution in TMD flake scrolling, an analog experiment was carried out. CVD-grown MoS_2 flakes were transferred from growth substrate to another bare SiO_2/Si substrate with a polymer-based method. In this process, the built-in tension in the flakes can be released because of the elastic polymer support [1]. Then the transferred sample was immersed into the ethanol solution for 30 minutes. Almost no scrolls were observed in the transferred sample after the procedures above, as shown in Supplementary Fig. 1a and b. The result indicated that the tension to scroll the flake was not induced by the immersing solution. It was observed that the immersing solution here was to intercalate into the flake and the substrate, thereby weakening the adhesion between them. This was crucial for built-in tension to initiate the flake scrolling, as analyzed in theory section (Supplementary Note 2). The existence of water was found to be indispensable to the intercalation. A strong interaction of hydrogen bond can be formed between water and the growth substrate (SiO_2/Si, Si_3N_4 or sapphire), which is stronger than the Van der Waals' force between TMD flakes (hydrophobic) and the substrate. Thus after being dropped on the sample, the water tends to substitute the flakes to contact with the substrate, leading to the intercalation between flakes and the substrate. Instantaneous intercalation happened to the samples when only water used. But the sharp intercalation often caused large pieces of TMD film peeled off together from the

substrate and floated in the water, giving rise to loosely curled, disordered nanoscrolls with large film unscrolled (Supplementary Fig. 1c). A moderate intercalation speed is required for the formation of high-quality nanoscrolls. Under a low intercalation speed, the substrate can act as a template to enable the flake to scroll straightly and tightly. Mixing water with water-soluble organic solvents, such as ethanol, methanol and dimethylformamide, was observed to slow down the intercalation. When only pure organic solvent was used, the intercalation proceeded quite slowly, and even cannot be observed. Optimizing the ratio of water and organic solvents in solution, high-quality scrolling of TMD flakes can be achieved. With the same concentration of aqueous solution, the scrolling speed slightly increased with the polarity of the organic solvent. High-quality scrolling can also be obtained with different solutions at the optimized ratio, as shown in Supplementary Fig. 2.

Supplementary Note 2. Energy analysis of the formation process of a MoS_2-NS

To understand the formation mechanism of a MoS_2-NS as a result of the release of elastic energy upon solvent intercalation, we present a comparative energy analysis of flat and scrolled MoS_2 sheets on a SiO_2/Si substrate.

For a stress-free MoS_2 sheet with length of l and width w adhered on a SiO_2/Si substrate, the potential energy composes of the interfacial adhesion energy $U_A = -\gamma_1 A$, where γ_1 is the areal adhesion energy density and $A = wl$ is the contact area between MoS_2 sheet and substrate, as well as the in-plane tensile energy of the MoS_2 sheet resulted from the mismatch in thermal strain as the MoS_2/substrate system is cooled from 720-825 °C to the room temperature, which is $U_I = E_{2D}[(\alpha_1 - \alpha_2)\Delta T]^2 wl/2$. Here E_{2D} is the in-plane tensile stiffness of monolayer MoS_2 sheet, α_1 and α_2 are the thermal expansion coefficients of MoS_2 and substrate, respectively, and ΔT is the change of temperature. The total potential energy (U_f) of the hybrid with a flat MoS_2 sheet on SiO_2/Si substrate is then

$$U_f = E_{2D}[(\alpha_1 - \alpha_2)\Delta T]^2 wl/2 - \gamma_1 wl \quad (1)$$

For a scrolled MoS_2 sheet on the SiO_2/Si substrate, the total energy (U) includes the self-adhesion energy of the MoS_2 sheet $U_S = -\gamma_2(l - l_s)$, and the out-of-plane bending energy (U_B) of MoS_2,

$$U_B = \frac{\pi D}{h}\ln\left(\frac{\sqrt{lh/\pi + r_0^2}}{r_0}\right),$$

where γ_2 is interlayer adhesion energy density between MoS_2 sheet itself, $l_s = 2\pi r_0$ is the critical length of cylindrical nucleus of the scroll, h is the interlayer spacing of MoS_2-NSs, and r_0 is the radius of the cylindrical nucleus, as shown in Supplementary

Fig. 5a, b. The total potential energy of the hybrid with MoS_2-NS on SiO_2/Si substrate is then

$$U = \begin{cases} \frac{\pi D w}{h} \ln\left(\frac{\sqrt{lh/\pi + r_0^2}}{r_0} \right) & (l < 2\pi r_0) \\ \frac{\pi D w}{h} \ln\left(\frac{\sqrt{lh/\pi + r_0^2}}{r_0} \right) - \gamma_2 w (l - 2\pi r_0) & (l \geq 2\pi r_0) \end{cases} \quad (2)$$

Using the typical measured values of parameters in our previous analysis, that are ΔT about 750 K, r_0 about 10 nm (from our experimental setup), h = 0.621 nm, γ_1 = 0.170 J m^{-2} [2], γ_2 = 0.140 J m^{-2} [3], E_{2D} = 122.30 N m^{-1} [4], D = 6.29 eV [4], $\alpha_1 = 7\times10^{-6}$ K^{-1} [5] and $\alpha_2 = 2.6\times10^{-6}$ K^{-1} [6]. For a flat sheet to bend into a scroll, a cylindrical nucleus has to be formed with a critical length of nucleation $l_s = 2\pi r_0$ (around 62.8 nm) as observed in our experiments, and an energy barrier E_b = 1.9 eV nm^{-1}, as calculated from supplementary equation 2.

As the solvent intercalation proceeds, the interlayer spacing between MoS_2 and substrate is increases and the adhesion energy γ_1 will decrease abruptly and we assume that γ_1 become zero (about 0 J m^{-2}) after the delamination.

We plot the total potential energy per width for a flat MoS_2 flake (blue line), a flat MoS_2 flake with intercalated solvent (red dash line), and a scrolled MoS_2 (red line), as a function of the contour length of sheets l (Supplementary Fig. 5c). Our energy analysis shows that there is a relatively low energy barrier of 0.3 nJ m^{-1} or 1.9 eV nm^{-1} for the scroll to nucleate below the critical length for scroll nucleation l_s, compared with the flat MoS_2 with intercalated solvent. As shown in the inset of Supplementary Fig. 5c, as the contour length of MoS_2 sheets exceeds 0.45 μm, the potential energy of

a flat MoS_2 with solvent intercalation (red dash line) is higher than the energy barrier to form scrolled MoS_2. This result indicates that the strain energy induced from the thermal mismatch of a flat MoS_2 is high enough to activate the formation of scrolls after solvent intercalation and decreasing the adhesion energy with substrate (Supplementary Fig. 5c). It should be remarked here that the conclusion holds on the condition that γ_1 becomes lower than γ_2 after solvent intercalation.

Supplementary Note 3. Analysis of the effect of compact scroll on FET mobility

We model the cross-section of a TMD-NS as an Archimedean spiral [7], as shown in Supplementary Fig. 15. The change of the scroll radius r along the length depends on the torsion angle θ expressed in polar coordinates:

$$r = a\theta + b \qquad (1)$$

where $a=t/2\pi$ is the spiral constant; t is the radius change per one full turn, approximately equal to the interlayer distance in the NS; b is the scroll radius at θ=0, i.e., the inner radius of the NS. As determined from TEM images, the t is approximately 0.6 nm and b is in the range of 5-20 nm for TMDs-NSs.

The arc length s of the Archimedean spiral is an integral by θ.

$$s = \int_0^s ds = \int_0^\theta \sqrt{(dr)^2 + (r\,d\theta)^2} \qquad (2)$$

Substitution of supplementary equation (1) into supplementary equation (2) yields

$$s = \int_0^\theta \sqrt{a^2 + (a\theta + b)^2}\,d\theta \qquad (3)$$

Based on the integral formula, the supplementary equation (3) can be computed as

$$s = \frac{a\theta + b}{2a}\sqrt{a^2 + (a\theta + b)^2} + \frac{a}{2}\ln\left[b + a\theta + \sqrt{a^2 + (a\theta + b)^2}\right] - \frac{b}{2a}\sqrt{a^2 + b^2} - \frac{a}{2}\ln\left(b + \sqrt{a^2 + b^2}\right) \qquad (4)$$

As shown in Supplementary Fig. 15, a TMD flake scrolled into a NS along one edge. The conduction channel width $W1$ for the TMD flake is just the arc length s of the NS spiral, while the conduction channel width $W2$ for the NS is equal to its diameter $2r$. Thus, the ratio of the conduction channel widths before and after scrolling can be described as

$$\frac{W1}{W2} = \frac{s}{2r} \qquad (5)$$

Substitution of supplementary equations (1) and (4) into supplementary equation (2) yields

$$\frac{W1}{W2} = \frac{1}{4a}\sqrt{a^2 + (a\theta + b)^2} + \frac{a}{4(a\theta + b)}\ln\left[b + a\theta + \sqrt{a^2 + (a\theta + b)^2}\right] - \frac{b}{4a(a\theta + b)}\sqrt{a^2 + b^2} - \frac{a}{4(a\theta + b)}\ln\left(b + \sqrt{a^2 + b^2}\right) \qquad (6)$$

Given that a is relatively small value of ~0.095, the supplementary equation (6) is simplified to

$$\frac{W1}{W2} = \frac{\theta}{4} - \frac{b^2}{4a(a\theta + b)} + \frac{b}{4a} \qquad (7)$$

Assuming that the flake rolled by full turns with $\theta=2n\pi$ (n, the scroll turns, i.e., layer number in the NS), the dependence of the ratio of the conduction channel widths on the scroll turns can be expressed as follows:

$$\frac{W1}{W2} = \frac{n\pi}{2} - \frac{b^2}{4a(2an\pi + b)} + \frac{b}{4a} \qquad (8)$$

Supplementary References

1. Amani, M. et al. High luminescence efficiency in MoS_2 grown by chemical vapor deposition. *ACS Nano* **10**, 6535−6541 (2016).

2. Deng, S. et al. Adhesion energy of MoS_2 thin films on silicon-based substrates determined via the attributes of a single MoS_2 wrinkle. *ACS Appl. Mater. Interfaces* **9**, 7812–7818 (2017).

3. Li, H., Wang, J., Gao, S. et al. Superlubricity between MoS_2 monolayers. *Adv. Mater.* **29**, 1701474 (2017).

4. Gao, E., Xu, Z. Thin-shell thickness of two-dimensional materials. *J. Appl. Mech.* **82**, 121012 (2015).

5. Sevik, C. Assessment on lattice thermal properties of two-dimensional honeycomb structures: Graphene, *h*-BN, *h*-MoS_2, and *h*-$MoSe_2$. *Phys. Rev. B* **89**, 035422 (2014).

6. Calizo, I., Balandin, A. A., Bao, W., Miao, F., Lau, C. N. Temperature dependence of the Raman spectra of graphene and graphene multilayers. *Nano Lett.* **7**, 2645–2649 (2007)

7. Krasilin, A. A. & Gusarov, V. V. Energy of formation of chrysotile nanotubes. *Russ. J. Gen. Chem.* **84**, 2359–2363 (2014).
