# Supplementary material for: Rolling up transition metal dichalcogenide nanoscrolls via one drop of ethanol
Source: Nat Commun. 2018 Apr 3;9:1301. doi: 10.1038/s41467-018-03752-5 (PMC5883047; doi:10.1038/s41467-018-03752-5)
Supplement: Supplementary file 2 — Description of Additional Supplementary Files(DOCX 14 kb) [file 41467_2018_3752_MOESM2_ESM.docx]

**Description of Additional Supplementary Files**

File Name: Supplementary Movie 1

Description: Supplementary Movie 1 shows the process of rolling up CVD-grown MoS2 flakes with one drop of ethanol solution.
